# Individualized Supplementation of Immunoactive Micronutrients and Severity of Upper Respiratory Infection Symptoms—A Randomized Intervention Study

**DOI:** 10.3390/nu16101400

**Published:** 2024-05-07

**Authors:** Melanie Haas, Beate Brandl, Laura Schinhammer, Thomas Skurk

**Affiliations:** 1 ZIEL–Institute for Food and Health, Core Facility Human Studies, Technical University Munich, Gregor-Mendel-Straße 2, 85354 Freising, Germany; 2 School of Medicine and Health, Technical University of Munich, Ismaninger Straße 22, 81675 Munich, Germany

**Keywords:** dried blood spots, upper respiratory tract infections, personalized nutrition, vitamin D, selenium, zinc, WURSS-21

## Abstract

Certain micronutrients exhibit immunomodulatory effects. However, no intervention has yet investigated the effect of individualized supplementation on the severity of upper respiratory tract infections (URIs). Therefore, we investigated whether a personalized supplementation moderates the incidence and severity of URI. Selenium, zinc, and vitamin D were measured in dried blood spots from 59 healthy participants. Accordingly, a personalized supplement was provided with or without the respective micronutrients. We used WURSS-21 questionnaires to assess the disease status. The blood values converged during the intervention and micronutrients no longer differed between treated and untreated volunteers at the end of the intervention period. The incidence and severity of the illness did not significantly differ between the groups. However, when analyzing the WURSS-21 scores by the intention to treat, the initially randomized treatment arm revealed a significantly higher score than the placebo arm. Upon acute administration, individualized combinations of selenium, zinc and vitamin D do not reduce the number, or contribute to a milder course of URIs. Therefore, supplementation in acute infectious situations seems questionable. Further studies must address the habitual diet in more detail, to better understand the impact of individual micronutrient status on the prevention of URI.

## 1. Introduction

A precisely tuned immune system is crucial to human health, as it protects organisms against noxious factors, like pathogens and foreign substances. Nutrition is a major exogenous factor that modulates immune function [1]. To ensure the proper function of immune cells and physical barriers, the immune system needs multiple specific nutrients, which play synergistic roles at every stage of the immune response in humans [2,3]. Several vitamins (D, A, C, E, folate, B6, B12) and minerals (zinc, iron, copper, and selenium) were documented to be essential for an optimal immune balance [4,5]. On the other hand, the mechanistic roles of micronutrients regarding immune function have been well described recently. Some of the micronutrients, like magnesium and Vitamin E, have accepted European health claims with an immune-modulatory context [6].

Especially since the outbreak of the Coronavirus pandemic, but also before, some micronutrients have been researched more extensively. In particular, vitamin D, selenium, and zinc have been discussed with regard to their effects on the immune system [7,8,9,10,11,12,13]. Vitamin D acts as an immune system modulator, preventing excessive expression of inflammatory cytokines and increasing macrophages’ ‘oxidative burst’ potential. Additionally, it stimulates the production of antimicrobial peptides, which are crucial for protecting the respiratory tract from infections [7,12,14]. Observational studies have indicated a correlation between low blood concentrations of 25-hydroxyvitamin D3, the precursor of calcitriol, which is the active form of vitamin D, and viral respiratory tract infections, such as URIs [15,16]. Vitamin D levels exhibit seasonal variability due to fluctuations in sunlight exposure, influencing cutaneous synthesis of the vitamin. Reduced sunlight exposure during winter, or in regions with limited sunlight, can lead to lower vitamin D levels [17]. Therefore, when assessing vitamin D status and prescribing supplementation, accounting for these seasonal biases is crucial.

The association between zinc deficiency and immune dysfunction has already been established 50 years ago, and since then, extensive research has been conducted in this field [18]. Zinc is involved in the regulation of the innate and adaptive immune response. It plays an essential role in lymphocyte formation, activation, and maturation, as well as in intercellular communication via cytokines and the innate host defense [13,18,19]. Insufficient zinc levels can result in thymus atrophy, lymphopenia, and impaired cellular and antibody-mediated immune responses [19]. Moreover, zinc deficiency has been linked to increased susceptibility to infections and delayed wound healing [20].

Selenium, which is also an essential micronutrient for human health, may contribute to a reduced immune function, as well as some cancers and viral diseases, in cases of measurable deficiency [11]. Animal models and epidemiological studies have provided evidence that low selenium levels can facilitate genetic mutations and increase the virulence of specific viruses, such as coxsackievirus, poliovirus, and murine influenza [21]. In addition to its role in immune function, selenium has been implicated in protecting against oxidative stress and reducing inflammation, further emphasizing its significance in maintaining overall health [22].

Yet, the relationship between immunoactive micronutrient supplements and their effect on the occurrence and severity of respiratory infections remains inconclusive. Some studies suggest that standardized intake of micronutrients can reduce the occurrence of respiratory infections [7,8,9,10,11,12,13], while others report no significant effect [5,15,16,23,24]. In particular, studies with older people, and/or those with micronutrient deficiencies, have demonstrated that micronutrient supplementation can reduce the incidence and severity of URIs [10,11,12,25]. However, for younger, otherwise healthy individuals, these benefits have not yet been consistently shown [5]. Furthermore, no studies have explored the potential benefits of personalized supplementation according to the individual micronutrient status.

Hence, the primary objective of our study was to investigate whether personalized supplementation, based on individual blood levels of selenium, zinc, and vitamin D, could moderate the occurrence and severity of URIs. To assess this, we used the Wisconsin Upper Respiratory Symptom Survey (WURSS-21), a standardized questionnaire for measuring URI symptoms [26,27]. In addition, our study aimed to address a gap in the existing research by examining the efficacy of personalized micronutrient supplementation in young, healthy individuals, where previous studies have been limited. By tailoring supplements to participants’ micronutrient blood status on an individual basis, we aimed to determine whether this approach could yield more significant improvements in immune function and respiratory health, compared to standardized supplementation protocols.

## 2. Materials and Methods

The procedure for the intervention was followed in accordance with the ethical standards from the Helsinki Declaration, and was approved 25 February 2021 by the ethics committee of the School of Medicine of the Technical University of Munich, Germany (75/21 S). All study participants provided online approval. The study was registered in the German Clinical Trials Register (DRKS): DRKS00023567.

### 2.1. Study Participants

For the intervention study cohort, in total, 94 healthy adults aged 18–65 years were recruited via announcements on social media (LinkedIn, Facebook, Instagram) and local newspaper advertisements. Power calculation was performed using G-power 3.1, assuming a Cohen effect size of 0.8, alpha error of 0.05, power of 0.9, and a dropout rate of 10%. A total of 62 study participants were then determined (28 per arm + 10%).

Participant enrollment started on April 23rd, 2021 and continued until 23 November, 2021. Participants were thoroughly informed about the objectives and scope of the planned intervention study via the online platform www.medico-health.de (3 April 2024). Detailed instructions were provided, prompting participants to register online and complete the comprehensive screening questionnaire aimed at evaluating their eligibility for participation in the study. Exclusion criteria were: age < 18 years, regular intake of supplements with vitamin D, selenium, zinc, and/or omega-3/omega-6 fatty acids, known intolerances to carrier substances of the supplement (guar gum, apple powder, potato starch), intention to go on vacation in southern climates (increased exposure to sunlight), and planned changes in dietary behavior (e.g., conversion to vegetarianism or veganism). If they met the inclusion criteria, they were directed to review the participant information. Subsequently, participants could provide their consent to participate by actively selecting a checkbox.

**Figure 1:**
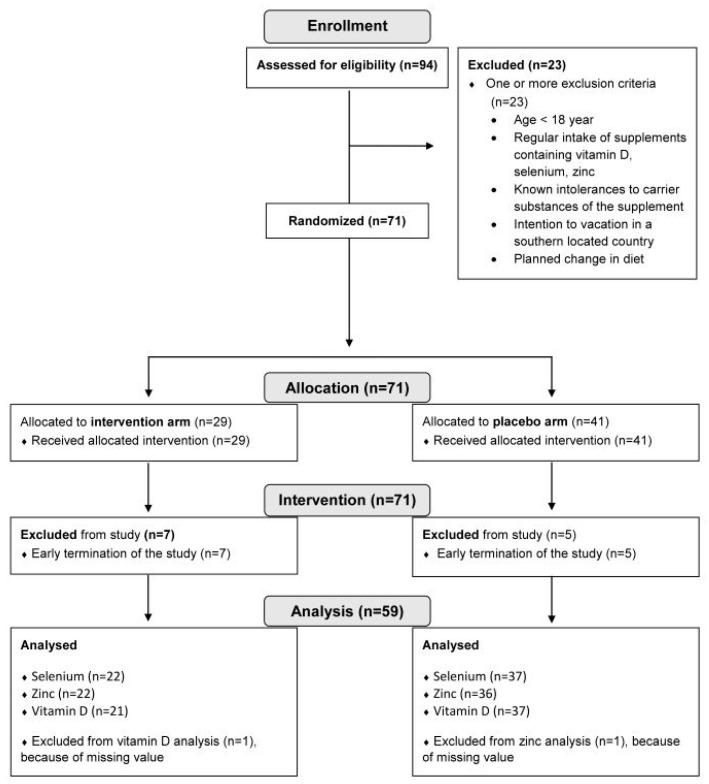
Intervention study participant flow diagram.

Randomization was performed online when participants registered for the study. Allocation to the study arms was carried out using a Phyton script with Phyton Random Library, based on the Mersenne Twister (Phyton version 3.6.8). Furthermore, a balancing feature, aiming for an equal group size, was used. Figure 1 depicts the participant flow chart of the intervention study.

The study was setup strictly under free-living conditions, therefore an adjustment of diet between the groups was not performed. In particular, with regard to vitamin D, selenium, and zinc, we believe that dietary adjustment was not necessary, as we saw only minor influences of different dietary variants in this respect. In addition, dietary habits were assessed and individuals with atypical patterns would have been excluded from the study.

### 2.2. Study Design

This was a randomized, double-blind, placebo-controlled intervention study. At the beginning of the study, all participants were asked to collect capillary blood from their fingertips with a home test kit for dried blood spots (DBSs; Whatman protein saver cards, cytiva, Marlborough, MA, USA) on their own. The DBSs were then sent to a laboratory (Vitas AS, Oslo, Norway) for analysis. Based on the DBS analysis, participants in the treatment arm received micronutrient supplementation with individual amounts of selenium, zinc, and/or vitamin D. Threshold blood levels for initiating supplementation were determined according to an algorithm from LOEWI^®^ (Munich, Germany) and were 0.14 µg/mL for selenium, 7 µg/mL for zinc, and 95 nmol/L for vitamin D. Accordingly, participants were randomized to the treatment groups and received either one, two, three, or no micronutrients. The placebo arm received only carrier material as a supplement. However, participants from the treatment arm who did not receive any micronutrients in the supplement due to blood levels were subsequently also analyzed as receiving “placebo”, compared to the “treatment” group who received either one, two, or all three micronutrients.

**Figure 2:**
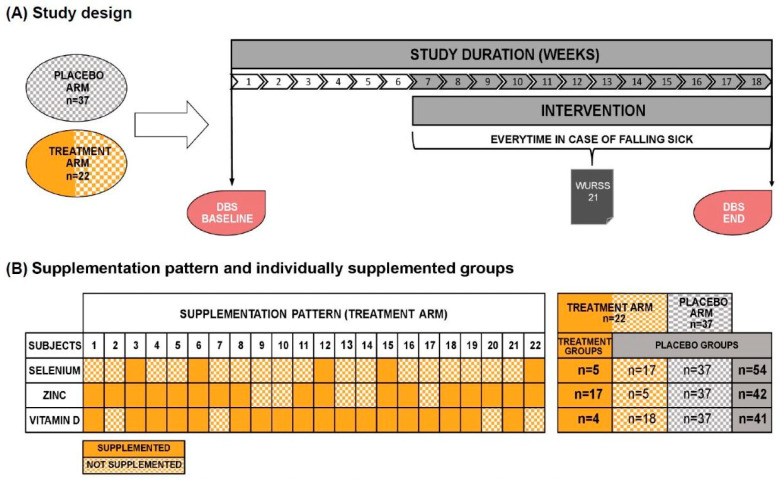
(**A**) Study design of the intervention study. Dried blood spots (DBSs) were sampled at the beginning and the end of the study. The whole study duration was approximately 18 weeks, of which the intervention lasted 12 weeks. WURSS-21 questionnaires were filled out in each case of falling sick. (**B**) Supplementation pattern of the treatment arm: full orange boxes indicate active treatment for the respective micronutrient; The orange-checkered boxes indicate no treatment and are, therefore, considered as receiving placebo for the respective micronutrient. Thus, each of the three placebo groups (selenium, zinc, vitamin D) appears as a sum of the other placebo arm (grey-checkered), together with the untreated participants from the treatment arm (orange-checkered). “Arm” refers, therefore, to the original randomization, whereas “groups” takes the real treatment into account.

Figure 2 summarizes the study design, the originally randomized study arms, and the split into individually supplemented groups. The treatment arm is colored two-tone, orange, and orange-checkered. Full orange indicates supplementation with the respective micronutrient (treatment group), whereas volunteers appearing as orange-checkered received no supplementation due to blood levels above the “critical supplementation level”. Consequently, we combined participants with an orange-checkered pattern from the treatment arm with the volunteers from the placebo arm (grey-checkered), now referred to as the placebo group (fully grey).

The average time period between DBS analysis and the start of supplementation was six weeks, to allow production of the personalized supplements. The intervention period was 12 weeks. The Wisconsin Upper Respiratory Symptom Survey-21 (WURSS-21) questionnaire was filled out in every case of falling sick during the study phase. Every WURSS-21 questionnaire was filled out on seven consecutive days. At the end of the intervention period, the participants provided another DBS sample.

### 2.3. Dried Blood Spot Analysis

After inclusion in the study, a home test kit with a detailed explanation for collecting DBSs was sent from the Core Facility Human Studies of the ZIEL-Institute for Food and Health, Freising, Germany, to the participants. Participants independently collected DBSs from their fingertips at the beginning of the study with a home test kit and after the intervention period. DBSs were sent to Vitas AS, Norway for the analysis of selenium [µg/mL], zinc [µg/mL], vitamin D [nmol/L], iron [µg/mL], magnesium [µg/mL], omega-3 fatty acids, and omega-6 fatty acids as a % of fatty acid methyl ester (FAME). The personalized micronutrient supplement was produced according to the blood profiles of selenium, zinc, and vitamin D.

### 2.4. Study Product

The study product, provided by LOEWI^®^, was a powder consisting of carrier material guar gum, apple powder, and potato starch. The product had to be taken daily during the complete intervention period (5 g). The placebo groups received the carrier material without micronutrients, whereas the treatment groups received the powder with an individually calculated amount of selenium, zinc and vitamin D. Only nutrients below a defined threshold were added (see above). Figure 2B shows the supplementation pattern and the individually supplemented groups. Selenium was supplemented in a range of 51.0 µg to 97.5 µg (mean = 66.9 µg), zinc from 7.2 µg to 26.7 µg (mean = 4.5 µg), and vitamin D from 22.5 µg to 97.5 µg (mean = 55.0 µg) per day. Accordingly, the amounts of supplemented micronutrients did not exceed the official national recommendations.

### 2.5. The Wisconsin Upper Respiratory Symptom Survey

We used the WURSS-21 [26,27] and the four additional symptoms; dry cough, fever, olfactory or taste disorder, and limb pain, to evaluate the number of episodes and the severity of URI during the intervention period. For each case of illness during the supplementation, participants were asked to fill out a WURSS-21 for seven consecutive days. Symptoms were ranked from 0 (no symptom) to 7 (severe symptom). Daily WURSS summary scores were calculated by adding up the scores (0–7) from each of the 14 symptoms. The day with the highest WURSS score was used to evaluate disease severity (WURSS_max_). A URI was defined by a WURSS_max_ sum score of 7 or higher. The highest possible value for one day would be 105.

### 2.6. Data Analysis and Statistics

Data were analyzed in the R programming environment and GraphPad Prism 10. Results are presented as mean ± SD, unless otherwise indicated. *p*-values < 0.05 were regarded as statistically significant. The Shapiro–Wilk test and quantile–quantile plots were used to test normal distribution of data. According to distribution, either the *t*-test or Wilcoxon test were applied to assess differences in the cohorts. To assess differences within the placebo and treatment groups between baseline and the end of the intervention period, the paired *t*-test was applied. One participant was excluded from the zinc and vitamin D analysis because of missing values due to low blood volume on the DBS filter paper.

## 3. Results

### 3.1. Participant Characteristics

In total, 94 volunteers were screened for our inclusion and exclusion criteria. Participant flow chart is shown in Figure 1. For analysis, the study cohort finally encompassed 59 participants, with 37 subjects in the placebo arm and 22 subjects in the treatment arm. As shown in Figure 2B and Table 1, the individually supplemented groups for selenium, zinc, and vitamin D were as follows: selenium (treatment group n = 5, placebo group n = 54), zinc (treatment group n = 17, placebo group n = 42), vitamin D (treatment group n = 18, placebo group n = 41). The placebo groups encompassed the placebo arm (randomized), plus the participants from the treatment arm without a deficit of the respective micronutrients. The treatment groups encompassed all participants with deficiencies for the respective micronutrient from the treatment arm. Table 1 depicts the study participants’ baseline characteristics and the division of the study arms into groups receiving the respective supplements.

### 3.2. Effect of Personalized Supplementation on the Micronutrient Status

**Figure 3:**
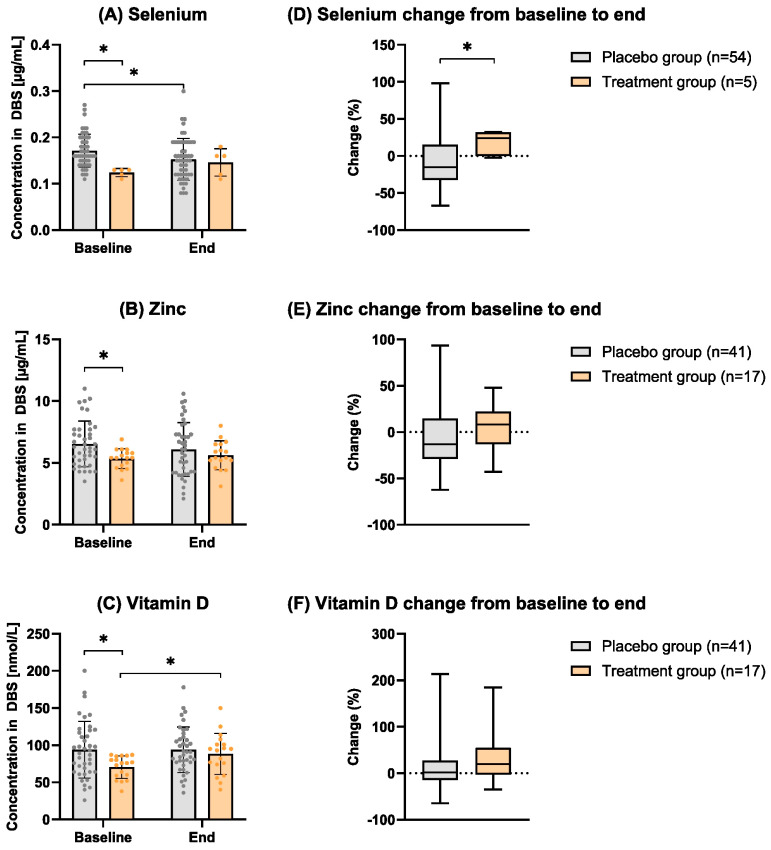
Micronutrient status between baseline and after 12 weeks of the intervention ((**A**–**C**), left) and the corresponding changes in % ((**D**–**F**), right). Comparison of the placebo and treatment group at baseline and at the end (according to distribution: *t*-test or Wilcoxon test). For changes of baseline and final values within the study group, a paired t-test was used. Bar plots with individual values (**A**–**C**), the bars represent the mean with SD. Box plots (**D**–**F**) are in the style of Tukey, the line represents the median one participant was excluded from the zinc and vitamin D analysis because of missing values due to low blood volume on the DBS filter paper. * = *p*-values < 0.05 were regarded as statistically significant.

In the following, volunteers in the intervention arm who did not receive any micronutrient treatment according to their baseline blood levels are analyzed together with the originally randomized placebo arm as one group. Figure 3 and Table 2 show the effect of individual supplementation with selenium, zinc, and vitamin D. At baseline, the placebo and treatment groups significantly differed in terms of the status of all three micronutrients. As expected, the placebo group showed significantly higher levels than the treatment group (*p*-value_selenium_ = 0.002, *p*-value_zinc_ = 0.024, *p*-value_vitamin D_ = 0.017). After the supplementation period of 12 weeks, the differences between the placebo and treatment group were no longer significant (*p*-value_selenium_ = 0.660, *p*-value_zinc_ = 0.946, *p*-value_vitamin D_ = 0.628). Thus, at the end of the study duration, the values of the different treatment groups adjusted to those of the untreated placebo groups (and Table 2). Although, all blood levels of single micronutrients in the supplemented group seemed to increase over time, but only vitamin D reached statistical significance after 12 weeks compared to the baseline levels (*p*-value = 0.015). Analysis of the percentage change in blood micronutrient levels from the beginning to the end of the study (Figure 3D–F) revealed a significant improvement in selenium levels between the placebo and treatment groups (*p*-value = 0.048). Although zinc and vitamin D exhibited a more substantial positive change in the treatment group compared to the placebo group, this difference did not reach statistical significance.

### 3.3. Individual Supplementation with Micronutrients Does Not Reduce Frequency or Moderate Severity of URI

During the entire study duration of seven months, a total of 16 URIs occurred in the study cohort; seven in the placebo arm and nine in the treatment arm. Only one participant in the placebo group reported a URI lasting more than one week. The highest WURSS_max_ reported was 67 by, a participant in the treatment group, the lowest WURSS_max_ was 11 and was reported by the placebo group. A summary of URI cases and WURSS_max_ scores in the individual supplemented groups and the study arms are provided in the Table 3. Interestingly, WURSS_max_ did not significantly differ in the single supplemented treatment and placebo groups, but the treatment and placebo arms, analyzed according to the initial intention to treat, show significant differences in the severity scores (*p*-value = 0.045).

We observed that, in most participants, the highest WURSS scores were recorded during the beginning to the middle of the seven days of recording the illness. However, from the fourth day onwards, symptoms started to decrease in almost all participants. On the last of the seven days of recording the WURSS score, only three participants had a WURSS_max_ above 7, which was indicative of being ill.

The most prominent symptoms reported during URI episodes were a plugged nose (with a total sum of all evaluation scores over a week of 257), feeling tired (sum of all evaluation scores over a week: 253), and coughing (sum of all evaluation scores over a week: 216). Conversely, the least pronounced symptoms included dry cough (sum of all evaluation scores over a week: 129), chest tightness (sum of all evaluation scores over a week: 125), and sneezing (sum of all evaluation scores over a week: 120).

## 4. Discussion

Despite various attempts in the past to supplement micronutrients as an adjuvant treatment for infections, the data remain controversial, and there is still limited evidence that individual micronutrients can be used to prevent or ameliorate URI symptoms [5,7,13,15,16,23]. Therefore, we aimed to investigate if a combination of selected immunoactive micronutrients (selenium, zinc, and vitamin D) with a personalized supplementation strategy, based on individual blood levels in healthy young volunteers, could be effective to reduce the frequency and severity of URIs. To our knowledge, this is the first study with such an approach.

First, we could see that supplementation over 12 weeks, in line with existing national recommendations [28,29,30], was able to alter relatively low blood levels of the selected micronutrients. The blood values became similar between the groups and the previously observed significance for selenium, zinc, and vitamin D disappeared between the groups after 12 weeks of supplementation. Within the treatment group, we observed a significant increase in vitamin D between baseline and final blood values. It is important to consider seasonal variations when administering vitamin D supplementation [17]. Our participants exhibited a relatively balanced seasonal distribution, as the participants’ supplementation periods were spread over the entire year. The blood levels for zinc and selenium within the treatment group did not reach statistical significance between baseline and the end of the study. However, the percentage change in selenium differs significantly between the placebo and the treatment group, and we attribute this difference to a statistical effect, as the selenium levels in the control group decreased significantly. In addition, a bias can be assumed due to the small number of effectively treated individuals (n = 5). Furthermore, we cannot preclude a selection bias, where participants are more likely to join the study if they have some interest in healthy eating and are less likely to be nutrient deficient, due to a balanced diet compared to the average population. In addition, we did not use pharmacological doses, as our intention was to use only moderate doses to correct deficiencies, which may require more time to be reflected in the results. We believe that selenium, zinc, and vitamin D are highly potent substances that should not be supplemented in higher doses over a longer period of time. It should also be noted that our intervention study was conducted in otherwise healthy individuals, who did not have clinically relevant deficiencies for the three micronutrients according to EFSA reference values [28,29,30]. Furthermore, the blood levels at which our participants received the respective supplementation were interpreted by an algorithm from LOEWI^®^, which is a continuum, and, therefore, the subjects received individual doses that did not follow a yes-no approach related to fixed thresholds.

Although we achieved comparable levels at the end of the trial with the individually composed nutritional supplement compared to the control group with normal blood values, we did not see an improvement in the occurrence or severity of URIs in the treated participants. Our results show that neither the occurrence, nor the severity of URIs during the study period differed between the intervention and placebo groups. The relatively low frequency of infection events is due, among other things, to the fact that the applicable contact restrictions of the Coronavirus pandemic prevented more URIs during the study period. Interestingly, when comparing the overall treatment group with the placebo group and analyzing the severity of illness according to intention to treat, the treatment group had significantly higher WURSS_max_ scores. This result may seem surprising at first glance, as there are several studies suggesting a beneficial effect of immunoactive micronutrient supplementation on URIs and COVID-19, especially vitamin D [7,31,32,33] and zinc [9,13]. For URIs, however, a positive effect was only shown in severe deficiency states [14,34]. Affirmingly, several other studies were also not able to show any significant amelioration in the frequency and severity of URIs when supplementing micronutrients [11,15,16,23,24]. For instance, a recent meta-analysis by Vlieg-Boerstra et al. found no association between the supplementation of proposed immunoactive nutrients, for example, vitamin D supplementation, and improved immune system response in the general European population [5]. Furthermore, the beneficial effects of supplementation reported in the literature have mainly been observed in studies of (older) individuals with low blood levels of micronutrients, and in longer-term supplementation studies [10,11,14,31]. In our study, all of our participants were younger and did not show signs of deficiency in the selected micronutrients, and it must be noted that we only used short-term supplementation, without pharmacological doses. Regarding the higher severity scores of the participants in the treatment arm, it is of course possible that the small number of participants may have biased our results. However, the findings show that even with adequate micronutrient status and supplementation with immunomodulatory micronutrients, people are not resistant to URIs and that other factors may contribute to the incidence and severity of URIs.

## 5. Conclusions

In conclusion, our results suggest that a strong immune defense does not depend solely on acute supplementation with individual micronutrients or a combination of vitamin D, zinc, and selenium. Rather, it appears to be a more complex interplay beyond the classical immunoactive micronutrients, pointing to the importance of a balanced diet providing all critical bioactives. Therefore, it is advisable to maintain a healthy, balanced diet that meets the recommended daily values of these nutrients, to ensure optimal functioning of physical barriers and immune cells. In addition, we believe it is important to consider other factors such as age, sex, comorbidity, and lifestyle. For future studies, we propose a more global approach that considers multiple factors, such as diet, when addressing immune system integrity.

## Figures and Tables

**Table 1:** Baseline characteristics.

	Treatment Arm	Placebo Arm	Overall
N ^1^	22	37	59
Sex (f/m), n	19/3	7/30	49/10
Age ^2^, years	26 ± 6	32 ± 13	30 ± 11
BMI, kg/m^2^	22.5 ± 3.8	22.8 ± 2.9	22.7 ± 3.3
Individually supplemented groups ^3^	Treatment group	Placebo group(untreated treatment arm + placebo arm)	Overall
Selenium, n	5	17 + 37	59
Zinc, n	17	5 + 37	59
Vitamin D, n	18	4 + 37	59

^1^ n = Study participants finally included in the analysis of intervention and observation cohort. ^2^ Age and BMI represent mean ± SD. ^3^ Individually supplemented groups are the division into groups according to the blood values in respective micronutrients, and refers to real treatment.

**Table 2:** Micronutrient status observed in DBSs at baseline and after 12 weeks (absolute changes and in %).

	Baseline ^1^	End	Change (%)
A: Selenium	[µg/mL]	[µg/mL]	
Placebo group (n = 54)	0.171 ± 0.036	0.152 ± 0.046	−6.03 ± 36.95
Treatment group (n = 5)	0.125 ± 0.009	0.147 ± 0.028	17.65 ± 16.32
*p*-value ^2^	0.002	0.660	0.048
B: Zinc	[µg/mL]	[µg/mL]	
Placebo group (n = 42)	6.53 ± 1.86	6.05 ± 2.15	−3.48 ± 36.75
Treatment group (n = 17)	5.34 ± 0.81	5.61 ± 1.16	6.90 ± 24.93
*p*-value	0.024	0.946	0.069
C: Vitamin D	[nmol/L]	[nmol/L]	
Placebo group (n = 41)	93.89 ± 38.16	93.89 ± 30.59	15.16 ± 58.17
Treatment group (n = 17)	70.46 ± 15.25	88.40 ± 27.61	31.45 ± 53.58
*p*-value	0.017	0.628	0.144

^1^ Data are presented as mean ± SD. ^2^ P-value describes significance between individually supplemented treatment groups and placebo groups. *p*-values < 0.05 were regarded as statistically significant. Significance was calculated using a Wilcoxon test or a *t*-test, according to the data distribution.

**Table 3:** Participants falling sick (n = 16) during study period.

		Placebo		Treatment		
		n of Falling Sick	WURSS_max_^1^	n of Falling Sick	WURSS_max_	*p*-Value ^2^
Study Arms		7	28.57 ± 11.84	9	43.11 ± 13.45	0.045
Individually supplemented groups	Selenium	14	36.71 ± 14.64	2	37.00 ± 18.38	ns
	Zinc	9	31.00 ± 12.70	7	44.14 ± 13.84	ns
	Vitamin D	8	33.37 ± 17.46	8	40.13 ± 10.72	ns

^1^ Data are presented as mean ± SD. ^2^ *p*-values < 0.05 were regarded as statistically significant. Significance was calculated using a Wilcoxon test or a *t*-test, according to the data distribution.

## Data Availability

Data described in the manuscript will be made available upon request.
